# A Novel Triple-Biomarker Score Predicts Mortality in Acute Pancreatitis

**DOI:** 10.14740/gr2120

**Published:** 2026-04-27

**Authors:** Rauf Agayev, Aliniyaz Mammadov, Emil Iskandarov, Farajullah Idrisov

**Affiliations:** a Rauf Agayev, Scientific Centre of Surgery Named After M.A.Topchubashov, Ministry of Health, AZ1122, Baku, Azerbaijan; b Aliniyaz Mammadov, 1st Department of Surgical Gastroenterology, Scientific Centre of Surgery Named After M.A.Topchubashov, Ministry of Health, AZ1122, Baku, Azerbaijan; c Training Division, The Administration of Regional Medical Divisions, AZ1065, Baku, Azerbaijan; d Farajullah Idrisov, 1st Department of Surgical Gastroenterology, Scientific Centre of Surgery Named After M.A.Topchubashov, Ministry of Health, AZ1122, Baku, Azerbaijan

**Keywords:** Acute pancreatitis, von Willebrand factor, TNF-α, C-reactive protein, Biomarker, Mortality, Prognosis

## Abstract

**Background:**

Predicting severe outcomes in acute pancreatitis (AP) remains a challenge. The interplay between endothelial dysfunction and systemic inflammation is pivotal in disease progression, but the combined prognostic value of their biomarkers is not well defined. The aims of this study were: 1) to define the interactions between endothelial dysfunction biomarkers and cytokines in patients with AP; 2) to evaluate the prognostic value of triple-marker model regarding prediction of survival in AP.

**Methods:**

In a prospective cohort of 100 AP patients, we serially measured biomarkers of endothelial dysfunction (vascular endothelial growth factor (VEGF), von Willebrand factor (vWF), endothelin, E-selectin) and inflammation (tumor necrosis factor-α (TNF-α), C-reactive protein (CRP), procalcitonin) on admission (day 0), day 1, and day 7. We employed correlation analyses, logistic regression, and receiver operating characteristic (ROC) analysis to assess their individual and combined ability to predict mortality.

**Results:**

A strong correlational network was observed, particularly among TNF-α, endothelin, E-selectin, CRP, and procalcitonin (all r > 0.75, P < 0.01). While VEGF correlated with TNF-α (P < 0.001), vWF did not show a significant bilateral correlation with it. Crucially, logistic regression revealed that the three-way interaction between vWF, TNF-α, and CRP was a significant predictor of mortality (P = 0.050). This triple-marker model demonstrated excellent predictive power for survival, with an area under the curve (AUC) of 0.861 in ROC analysis (P < 0.001). Kaplan–Meier analysis confirmed that patients with a high vWF–TNF-α–CRP interaction score had significantly lower survival rates (69.2% vs. 98.4%, P < 0.001).

**Conclusions:**

The combination of endothelial and inflammatory markers is a more powerful prognostic tool than any marker in isolation. The vWF–TNF-α–CRP interaction model effectively identifies AP patients at high risk of mortality, potentially enabling earlier targeted interventions. This underscores the critical role of the endothelial–inflammatory axis in determining AP outcomes.

## Introduction

The management of complicated acute pancreatitis (AP) requires strengthened healthcare services in specialized centers [1, 2], especially as its global incidence is increasing [3, 4]. According to the Atlanta classification, mild forms of the disease and acute severe forms resulting in necrosis of the glandular tissue are determined [5]. Despite significant advances in the diagnosis and treatment of AP, the unpredictable clinical course and high incidence of severe complications remain major clinical challenges. Current prognostic scoring systems (e.g. Ranson, BISAP, APACHE-II, SOFA), while valuable, lack the precision to reliably predict the critical transition from mild, self-limiting disease to severe necrotizing pancreatitis, underscoring the urgent need for more sensitive and dynamic biomarkers [6].

The main etiological factors are excessive alcohol intake and gallstone disease. Bile acids and alcohol induce the activation of digestive enzymes, leading to acinar damage, which disrupts normal tissue perfusion and results in cell damage, inflammation, and a cytokine storm [7]. Crucially, inflammation and coagulation are tightly linked through a reciprocal, self-amplifying feedback loop. Inflammatory cytokines such as tumor necrosis factor-α (TNF-α) and interleukin (IL)-6, released from activated immune cells, induce the expression of tissue factor (TF) on endothelial cells and monocytes, activating the extrinsic coagulation pathway while simultaneously reducing natural anticoagulant activity and impairing fibrinolysis [8, 9]. This promotes a prothrombotic state. Furthermore, TNF-α and IL-6 disrupt endothelial integrity, increasing vascular permeability. Activated endothelial cells respond by expressing adhesion molecules and releasing mediators like von Willebrand factor (vWF) and vascular endothelial growth factor (VEGF). vWF facilitates platelet adhesion and aggregation, while VEGF increases vascular permeability, worsening edema and tissue hypoxia [10, 11]. This hypoxia, in turn, induces further endothelial damage, stimulating the release of additional proinflammatory and procoagulant mediators and resulting in increased cytokine production (TNF-α, IL-6). This vicious cycle perpetuates inflammation, ischemia, and microvascular thrombosis, forming the core of the inflammation–endothelial dysfunction axis [12, 13].

While individual markers of inflammation and endothelial injury have been previously associated with AP severity, their isolated measurement has limited clinical utility due to overlap with mild disease and temporal variability. Given the complex interplay between these pathways, we hypothesized that measuring multiple markers simultaneously would better capture the multifactorial nature of AP progression than any single marker alone. We propose that the synergistic interaction between key players from both pathways may provide a more powerful prognostic signal.

Based on the pathophysiological roles of vWF (endothelial activation), TNF-α (pro-inflammatory cytokine), and C-reactive protein (CRP) (downstream acute phase reactant), we hypothesized a priori that their interaction would provide superior prognostic value. The additional markers were measured to validate the underlying endothelial–inflammatory network and to ensure that no alternative combination outperformed our hypothesized model.

The aims of this study were: 1) to define the interactions between endothelial dysfunction biomarkers and cytokines in patients with AP; 2) to evaluate the prognostic value of triple-marker model regarding prediction of survival in AP.

## Materials and Methods

### Study design and ethical approval

This prospective cohort study was performed at the Academic M.A. Topchubashov Scientific Surgery Center between 2019 and 2022. The study protocol was reviewed and approved by the Ethics Committee of the Public Legal Entity “Academic M.A. Topchubashov Scientific Surgery Center,” Ministry of Health of the Republic of Azerbaijan (Approval No. 6, September 17, 2019). This committee functions as the institutional review board for research involving human subjects in accordance with national regulations. Written informed consent was obtained from all participants prior to inclusion. The committee confirmed that the investigation involved no foreseeable risk of physical or material harm to the participants.

### Patient population

A total of 100 consecutive patients with AP were enrolled. Only adult patients (≥ 18 years) were included. No upper age limit was applied. Patients were enrolled consecutively between January 2019 and December 2022. The diagnosis of AP was established based on the presence of at least two of the following three criteria: 1) abdominal pain consistent with AP; 2) serum amylase and/or lipase activity ≥ 3 times the upper limit of normal; and 3) characteristic imaging findings on contrast-enhanced computed tomography [5].

### Exclusion criteria

Patients with pre-existing chronic hepatic or renal failure, active malignant disease, or significant cardiovascular or hematologic disorders were excluded from the study. Pregnant women and pediatric patients (< 18 years) were excluded from the study due to the different physiological responses and biomarker profiles in these populations.

### Study endpoint and subgroup classification

The primary endpoint was all-cause in-hospital mortality. To analyze biomarker dynamics in relation to disease severity, the patient cohort was stratified based on clinical course. We have explicitly aligned our classifications with the Revised Atlanta Classification (2012). The mild course group included patients with no organ failure and no local or systemic complications. The severe course group included patients with persistent organ failure (> 48 h), as defined by a Marshall score ≥ 2 for at least one of three organ systems (respiratory, cardiovascular, renal). This group was further divided into two subgroups: subgroup A and B: 1) Subgroup A (worsened course): patients who developed multiorgan dysfunction (MOD) and either required intensive care unit (ICU) admission or failed to show improvement in organ failure by day 7; 2) Subgroup B (improved course): patients who developed MOD but demonstrated clinical improvement and resolution of organ failure by day 7 with conventional therapy.

### Sampling and biomarker analysis

The blood was collected for the first time on admission (day 0), prior to confirmation of diagnosis, and later in day 3, and day 7 of hospitalization. Plasma was separated by centrifugation and stored at –80 °C until analysis.

#### Blood collection tubes

For plasma markers (VEGF, vWF, endothelin, E-selectin, procalcitonin), EDTA tubes (BD Vacutainer^®^) were used. For serum markers (TNF-α, CRP), serum separator tubes (SST, BD Vacutainer^®^) were used.

#### Processing time

All samples were centrifuged within 30 min of collection at 4 °C (1,500 × g for 15 min).

#### Storage during processing

Samples were kept on ice until centrifugation.

#### Storage duration

Samples were stored at –80°C for a maximum of 18 months (range: 2–18 months) before analysis. All assays were performed within the manufacturer’s stated stability period.

Plasma concentrations of endothelial dysfunction markers and procalcitonin were determined using commercial enzyme-linked immunosorbent assay (ELISA) kits (Cloud-Clone Corp., Katy, TX, USA). Serum CRP and TNF-α levels were measured using a latex turbidimetric immunoassay and a specific ELISA kit, respectively. Detailed specifications for all assay kits are provided in Table 1. All assays were performed in duplicate according to the manufacturer’s instructions.

**Table 1:** Specifications of Commercial Assay Kits Used for Biomarker Quantification

Biomarker	Kit name (supplier)	Catalog number	Sample type	Assay type	Detection range	Sensitivity
VEGF	Human VEGF165 ELISA Kit (Cloud-Clone Corp.)	SEB696Hu	Plasma	Sandwich ELISA	15.6–1,000 pg/mL	< 5.8 pg/mL
Endothelin-1	Human Endothelin 1 ELISA Kit (Cloud-Clone Corp.)	CEA482Hu	Plasma	Sandwich ELISA	6.17–500 pg/mL	< 2.71 pg/mL
vWF	Human vWF ELISA Kit (Cloud-Clone Corp.)	SEB696Hu	Plasma	Sandwich ELISA	15.6–1,000 pg/mL	< 5.8 pg/mL
E-selectin	Human SELE ELISA Kit (Cloud-Clone Corp.)	SEA029Hu	Plasma	Sandwich ELISA	39–2,500 pg/mL	< 17 pg/mL
Procalcitonin	Human PCT ELISA Kit (Cloud-Clone Corp.)	SEA689Hu	Plasma	Sandwich ELISA	31.2–2,000 pg/mL	< 12.4 pg/mL
TNF-α	Human TNF-α ELISA Kit (Vector-Best)	VE-ELH-TNFa	Serum	Sandwich ELISA	15.6–1,000 pg/mL	< 5.0 pg/mL
CRP	CRP LATEX Kit (Monlab Reagents)	3100	Serum	Latex turbidimetric immunoassay	0.2–20 mg/dL (2–200 mg/L)	< 0.2 mg/dL (2 mg/L)

All samples were collected, processed, and stored according to the manufacturers’ protocols and institutional ethical guidelines. Sensitivity values are given as the minimum detectable concentration according to the respective kit insert. For CRP, results are reported in both mg/dL and mg/L; conversion factor: 1 mg/dL = 10 mg/L. Catalog numbers and kit names correspond to the commercial products used at the time of the study. VEGF: vascular endothelial growth factor; vWF: von Willebrand factor; TNF-α: tumor necrosis factor-α; CRP: C-reactive protein; ELISA: enzyme-linked immunosorbent assay.

### Statistical analysis

Statistical analyses were performed using IBM SPSS Statistics v22.0. Continuous data are presented as mean ± standard error of the mean (SEM) or median (interquartile range (IQR)) based on distribution normality (assessed by Shapiro-Wilk test). Categorical data are expressed as frequencies and percentages. Group comparisons for continuous variables were made using the Student’s *t*-test or Mann–Whitney U test, as appropriate. Categorical variables were compared using the Chi-square test.

All patients were followed for 30 days post-admission or until death, whichever occurred first. Patients who were alive at 30 days were administratively censored at that time point. This fixed follow-up period ensures that censoring is non-informative, as it is determined by the study design rather than patient characteristics or outcomes.

The independent variables included the core biomarkers (vWF, TNF-α, CRP) measured at admission, along with key clinical covariates (e.g., age, sex). The performance of this model was evaluated using receiver operating characteristic (ROC) curve analysis. Correlation between continuous variables was assessed using Pearson or Spearman coefficients. A two-tailed P value < 0.05 was considered statistically significant.

To test the primary hypothesis, a triple biomarker score (TBS) was calculated for each patient on admission by multiplying the raw values of vWF (pg/mL), TNF-α (pg/mL), and CRP (mg/L) (TBS = vWF × TNF-α × CRP). This score was used as a continuous variable in a binary logistic regression model with in-hospital mortality as the dependent variable.

The predictive performance of the TBS for in-hospital mortality was evaluated using ROC curve analysis. The area under the curve (AUC) was calculated with 95% confidence intervals (CIs). The optimal cut-off value was determined using Youden’s index (J = sensitivity + specificity – 1), which identifies the point on the ROC curve maximizing the sum of sensitivity and specificity, thereby providing the best balance between true positive and false positive rates. Bootstrap resampling with 1,000 iterations was performed to validate the stability of the AUC and cut-off estimates.

## Results

### Patient characteristics and clinical outcomes

A total of 100 patients with AP were stratified by disease severity. The mild course group (n = 68) experienced a mortality rate of 4.4% (three patients). In contrast, the severe course group (n = 32) had a significantly higher mortality rate of 31.3% (10 patients). Within the severe course group, a post-hoc analysis revealed that all mortality events occurred in subgroup A (worsened course), which included patients who developed MOD or required ICU admission. No deaths occurred in subgroup B (improved course) or the remaining patients of the severe course group who did not meet subgroup A criteria (Table 2).

**Table 2:** Patient Characteristics and Clinical Outcomes Stratified by Disease Severity

Characteristic	Mild course group (n = 68)	Severe course group (n = 32)	Subgroup A: worsened (n = 17)	Subgroup B: improved (n = 15)
Age, years, mean ± SD (range)	48.0 ± 12.5 (19–82)	49.5 ± 11.8 (16–79)	50.0 ± 10.1 (26–79)	49.0 ± 13.8 (16–75)
Male sex, n (%)	30 (44.1)	16 (50.0)	8 (47.1)	8 (53.3)
Weight, kg, mean ± SD (range)	77.0 ± 15.0 (37–111)	79.0 ± 12.5 (53–106)	80.0 ± 10.5 (55–95)	78.0 ± 14.5 (53–106)
Height, cm, mean ± SD (range)	166.0 ± 8.5 (144–189)	163.5 ± 7.5 (147–178)	163.0 ± 8.0 (147–175)	164.0 ± 7.0 (156–178)
BMI, kg/m^2^, mean ± SD (range)	27.0 ± 4.5 (22–37)	29.5 ± 5.5 (22–44)	30.0 ± 5.0 (24–41)	29.0 ± 6.0 (22–44)
Hospital stay, days, mean ± SD (range)	8.56 ± 5.37 (1–31)	14 ± 7.83 (3–42)	18.53 ± 8.16 (7–42)	8.87 ± 2.56 (3–13)
ICU stay, days, mean ± SD (range)	1.62 ± 1.42 (1–6)	7.30 ± 8.60 (1–42)	9.81 ± 10.33 (1–42)	3.64 ± 2.73 (1–9)
Time from onset of symptoms to admission, days				
Mean ± SD	136.35 ± 19.68	55.69 ± 19.05	61.59 ± 29.51	49 ± 24.08
Median	30	9	7	14
IQR	358	25	25	67
Complications, n (%)				
Pancreatic necrosis	3 (4.41%)	9 (28.12%)	6 (35.29%)	3 (20%)
Pseudocyst	3 (4.41%)	3 (9.37%)	1 (5.88%)	2 (13.3%
Infected necrosis	10 (14.7%)	13 (40.62%)	8 (47.05%)	5 (53.3%)
Organ failure	8 (11.76%)	3 (9.37%)	1 (5.88%)	2 (13.3%)
Mortality, n (%)	3 (4.4%)	10 (31.3%)	10 (58.8%)	0 (0)

SD: standard deviation; IQR: interquartile range; BMI: body mass index; ICU: intensive care unit.

### Temporal trends of biomarkers stratified by survival status

**Figure 1:**
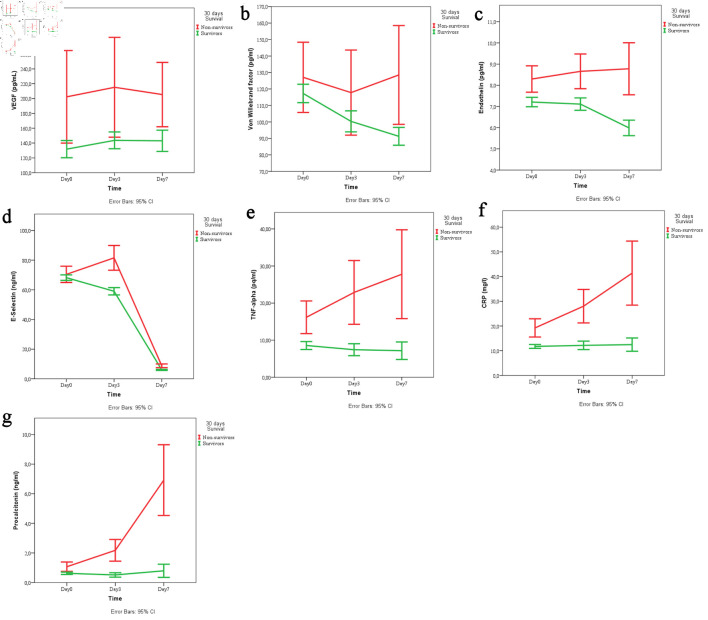
Temporal trends of (a) VEGF, (b) von Willebrand factor, (c) endothelin, (d) E-selectin, (e) TNF-α, (f) CRP, and (g) procalcitonin across the three time points (day 1, 3, 7) stratified by survival status (survivors vs. non-survivors). VEGF: vascular endothelial growth factor; TNF-α: tumor necrosis factor-α; CRP: C-reactive protein; CI: confidence interval.

To visualize the dynamic changes in biomarker levels over time, we plotted the mean values of all seven biomarkers at admission, day 3, and day 7 stratified by survival status (survivors vs. non-survivors). These temporal trends are presented in Figure 1 (a–g).

### Correlation between inflammatory and endothelial markers on admission

A cross-sectional analysis was performed to determine the relation between TNF-α and markers of endothelial dysfunction. The TNF-α on admission day was 9.55 ± 0.59 pg/mL. Spearman correlation analysis revealed a significant positive relationship between TNF-α and VEGF levels on admission (P < 0.001). An increase in TNF-α levels resulted with an increase in VEGF levels, which indicates the interaction of inflammatory and angiogenesis processes.

When examining the relationship between TNF-α and other endothelial function markers, a significant positive correlation was found with endothelin and E-selectin (r = 0.760; P < 0.05), but not with vWF levels (P > 0.05). As shown from Table 3, there was a positive relationship between TNF-α, VEGF, endothelin, and E-selectin.

**Table 3:** Correlation Matrix of Biomarkers on Admission (Adm) Day

Correlations									
			VEGF_Adm	vWF_Adm	End_Adm	E-Sel_Adm	TNF_Adm	CRP_Adm	Proc_Adm
Spearman’s rho	VEGF_Adm	Correlation coefficient	1.000	0.042	0.455**	0.205*	0.364**	0.236*	0.377**
		Sig. (two-tailed)		0.681	0.000	0.041	0.000	0.018	0.000
		N	100	100	100	100	100	100	100
	vWF_Adm	Correlation coefficient	0.042	1,000	0.039	0.177	–0.038	0.094	0.192
		Sig. (two-tailed)	0.681		0.703	0.078	0.709	0.354	0.056
		N	100	100	100	100	100	100	100
	End_Adm	Correlation coefficient	0.455**	0.039	1.000	0.169	0.684**	0.429**	0.598**
		Sig. (two-tailed)	0.000	0.703		0.093	0.000	0.000	0.000
		N	100	100	100	100	100	100	100
	E-Sel_Adm	Correlation coefficient	0.205*	0.177	0.169	1.000	0.211*	0.064	0.299**
		Sig. (two-tailed)	0.041	0.078	0.093		0.035	0.526	0.002
		N	100	100	100	100	100	100	100
	TNF_Adm	Correlation coefficient	0.364**	–0.038	0.684**	0.211*	1.000	0.276**	0.596**
		Sig. (two-tailed)	0.000	0.709	0.000	0.035		0.005	0.000
		N	100	100	100	100	100	100	100
	CRP_Adm	Correlation coefficient	0.236*	0.094	0.429**	0.064	0.276**	1.000	0.433**
		Sig. (two-tailed)	0.018	0.354	0.000	0.526	0.005		0.000
		N	100	100	100	100	100	100	100
	Proc_Adm	Correlation coefficient	0.377**	0.192	0.598**	0.299**	0.596**	0.433**	1.000
		Sig. (two-tailed)	0.000	0.056	0.000	0.002	0.000	0.000	
		N	100	100	100	100	100	100	100

Data are presented as Spearman correlation coefficient (r). The diagonal of 1.0 represents the correlation of each variable with itself. **Correlation is significant at the 0.01 level (two-tailed). *Correlation is significant at the 0.05 level (two-tailed). VEGF: vascular endothelial growth factor; vWF: von Willebrand factor; TNF: tumor necrosis factor; CRP: C-reactive protein; End: endothelin-1; E-Sel: E-selectin; Proc: procalcitonin; Sig.: significance.

### Biomarker correlations day 3

We examined the correlations between endothelial dysfunction markers, tissue cytokines and inflammatory mediators in patients with AP on the third day of treatment. Analysis of biomarker correlations on day 3 of hospitalization revealed a tightly interconnected network between mediators of endothelial dysfunction and systemic inflammation (Table 4).

**Table 4:** Correlation Matrix of Biomarkers on Day 3 of Hospitalization

Correlations									
			VEGF_3d	vWF_3d	End_3d	E-Sel_3d	TNF_3d	CRP_3d	Proc_3d
Spearman’s rho	VEGF_3d	Correlation coefficient	1.000	0.181	0.328**	0.426**	0.277**	0.333**	0.238*
		Sig. (two-tailed)		0.072	0.001	0.000	0.005	0.001	0.017
		N	100	100	100	100	100	100	100
	vWF_3d	Correlation coefficient	0.181	1.000	0.207*	0.137	0.255*	0.262**	0.209*
		Sig. (two-tailed)	0.072		0.038	0.174	0.011	0.009	0.037
		N	100	100	100	100	100	100	100
	End_3d	Correlation coefficient	0.328**	0.207*	1.000	0.402**	0.696**	0.562**	0.722**
		Sig. (two-tailed)	0.001	0.038		0.000	0.000	0.000	0.000
		N	100	100	100	100	100	100	100
	E-Sel_3d	Correlation coefficient	0.426**	0.137	0.402**	1.000	0.485**	0.543**	0.430**
		Sig. (two-tailed)	0.000	0.174	0.000		0.000	0.000	0.000
		N	100	100	100	100	100	100	100
	TNF_3d	Correlation coefficient	0.277**	0.255*	0.696**	0.485**	1.000	0.517**	0.642**
		Sig. (two-tailed)	0.005	0.011	0.000	0.000		0.000	0.000
		N	100	100	100	100	100	100	100
	CRP_3d	Correlation coefficient	0.333**	0.262**	0.562**	0.543**	0.517**	1.000	0.555**
		Sig. (two-tailed)	0.001	0.009	0.000	0.000	0.000	.	0.000
		N	100	100	100	100	100	100	100
	Proc_3d	Correlation coefficient	0.238*	0.209*	0.722**	0.430**	0.642**	0.555**	1.000
		Sig. (two-tailed)	0.017	0.037	0.000	0.000	0.000	0.000	
		N	100	100	100	100	100	100	100

Data are presented as Spearman correlation coefficient (r). The diagonal of 1.0 represents the correlation of each variable with itself. **Correlation is significant at the 0.01 level (two-tailed). *Correlation is significant at the 0.05 level (two-tailed). 3d: day 3; VEGF: vascular endothelial growth factor; vWF: von Willebrand factor; TNF: tumor necrosis factor; CRP: C-reactive protein; End: endothelin-1; E-Sel: E-selectin; Proc: procalcitonin; Sig.: significance.

### Temporal trends in biomarker levels

Serial measurements of all seven biomarkers were performed at admission, day 3, and day 7. Table 5 presents the complete longitudinal data for each biomarker stratified by the three clinically relevant subgroups: mild course (n = 68), worsened subgroup A (n = 17), and improved subgroup B (n = 15).

**Table 5:** Data for All Seven Biomarkers at Admission, Day 3, and Day 7, Stratified by the Three Clinically Relevant Subgroups (Mild Course, Worsened Subgroup A, Improved Subgroup B).

Subgroups		Admission							Day 3							Day 7						
		VEGF	vWF	End	E-Sel	TNF	CRP	Proc	VEGF	vWF	Endo	E-Sel	TNF	CRP	Proc	VEGF	vWF	Endo	E-Sel	TNF	CRP	Proc
Mild course (n = 68)	Mean	120.1985	116.0103	6.7050	67.2397	6.1394	10.8115	0.4509	136.7809	93.9397	6.3775	56.4074	4.0731	9.6190	0.2140	130.3191	86.0426	5.1137	47.9147	3.2999	8.6996	0.0896
	N	68	68	68	68	68	68	68	68	68	68	68	68	68	68	68	68	68	68	68	68	68
	SD	37.27671	22.64100	0.62950	8.72104	1.29862	2.67186	0.16683	37.85918	20.75935	0.46722	7.79302	0.83722	1.60439	0.09359	41.18839	17.14541	0.44807	6.57815	0.69896	1.28015	0.01165
	Min	35.00	61.00	4.80	35.30	4.12	6.98	0.12	40.00	59.00	4.02	30.20	3.04	6.03	0.06	50.40	56.00	3.93	25.40	2.51	5.98	0.05
	Max	260.00	173.00	7.82	79.20	11.96	15.20	0.91	270.00	165.80	7.23	68.20	9.97	13.96	0.42	288.70	154.00	6.18	59.80	7.40	12.03	0.11
Worsened (subgroup A, n = 17)	Mean	194.7706	122.0118	8.9129	72.7941	17.5571	20.5294	1.3029	209.7647	136.0706	9.4947	87.3529	28.4971	34.9235	2.6853	230.1529	141.0118	9.9376	99.3118	37.5529	51.6706	8.3018
	N	17	17	17	17	17	17	17	17	17	17	17	17	17	17	17	17	17	17	17	17	17
	SD	93.57457	35.95207	0.29995	8.68083	5.15605	4.86181	0.36467	96.28630	45.88323	0.52547	8.13297	9.87700	6.66638	0.67861	94.56945	45.16745	0.93069	8.18305	15.77910	12.82803	1.91778
	Min	50.00	66.30	8.30	50.30	6.20	10.00	0.52	53.20	59.00	8.70	70.00	8.20	20.20	0.65	55.30	77.00	8.60	85.00	15.00	25.00	1.40
	Max	470.00	176.00	9.28	85.00	28.00	29.80	1.96	508.00	192.00	10.60	100.00	40.00	48.00	3.90	520.00	224.00	11.50	109.70	62.00	67.00	9.98
Improved (subgroup B, n = 15)	Mean	174.2200	126.1067	8.5033	69.3600	15.9127	12.4467	1.0227	161.9333	104.0000	9.0807	58.3467	12.1313	11.7260	0.8533	155.9600	90.8467	7.8953	45.3267	8.0407	10.2000	0.7820
	N	15	15	15	15	15	15	15	15	15	15	15	15	15	15	15	15	15	15	15	15	15
	SD	88.63886	36.38336	0.36233	8.32104	5.58683	2.72341	0.28702	96.54062	33.35583	0.55595	9.65970	3.64873	2.96259	0.15003	85.00817	22.18065	0.55575	5.88818	2.49135	2.58706	0.12712
	Min	46.00	61.00	8.00	46.00	6.10	8.00	0.52	39.00	59.00	8.20	39.00	5.20	8.00	0.40	38.00	56.00	6.80	35.00	4.00	7.80	0.40
	Max	460.00	173.00	9.20	78.00	24.90	20.00	1.60	430.00	166.00	9.90	68.00	19.00	19.00	1.10	415.00	154.00	8.62	61.00	12.90	18.00	0.98
Total	Mean	140.9790	118.5450	7.3501	68.5020	9.5464	12.7088	0.6815	152.9610	102.6110	7.3129	61.9590	9.4339	14.2368	0.7300	151.1370	96.1080	6.3510	56.2640	9.8340	16.2297	1.5895
	N	100	100	100	100	100	100	100	100	100	100	100	100	100	100	100	100	100	100	100	100	100
	SD	66.53957	27.53462	1.09912	8.82113	5.91478	4.76298	0.41528	67.32353	32.14688	1.45875	14.10197	10.06817	9.96256	0.96190	70.63113	31.91012	1.98531	20.71782	14.25475	16.99393	3.15873
	Min	35.00	61.00	4.80	35.30	4.12	6.98	0.12	39.00	59.00	4.02	30.20	3.04	6.03	0.06	38.00	56.00	3.93	25.40	2.51	5.98	0.05
	Max	470.00	176.00	9.28	85.00	28.00	29.80	1.96	508.00	192.00	10.60	100.00	40.00	48.00	3.90	520.00	224.00	11.50	109.70	62.00	67.00	9.98

SD: standard deviation; Min: minimum; Max: maximum; VEGF: vascular endothelial growth factor; vWF: von Willebrand factor; TNF: tumor necrosis factor; CRP: C-reactive protein; End: endothelin-1; E-Sel: E-selectin; Proc: procalcitonin.

Notably, the inflammatory cytokine TNF-α demonstrated strong positive correlations with the endothelial factors endothelin (r = 0.779) and E-selectin (r = 0.754), as well as with the systemic inflammatory markers CRP (r = 0.829) and procalcitonin (r = 0.879). Similarly, the endothelial markers endothelin and E-selectin were strongly correlated with each other (r = 0.585) and with both CRP and procalcitonin (r values ranging from 0.772 to 0.791).

VEGF and vWF also showed significant positive correlations with this core network, though the relationships were generally more moderate in strength. The strongest correlation was observed between the classic inflammatory markers CRP and procalcitonin (r = 0.895, P < 0.01).

This comprehensive correlation analysis confirms a robust and synergistic relationship between endothelial cell activation and the systemic inflammatory response during AP.

The effect of the three-way interaction between endothelial dysfunction markers (VEGF, von Willebrand, endothelin, E-selectin) and TNF and CRP/procalcitonin on survival/mortality was studied through logistic regression analysis. The analysis of the omnibus tests of model coefficients showed that the model was statistically significant (Chi-square = 27.650, P = 0.001).

### TBS and mortality prediction

**Figure 2:**
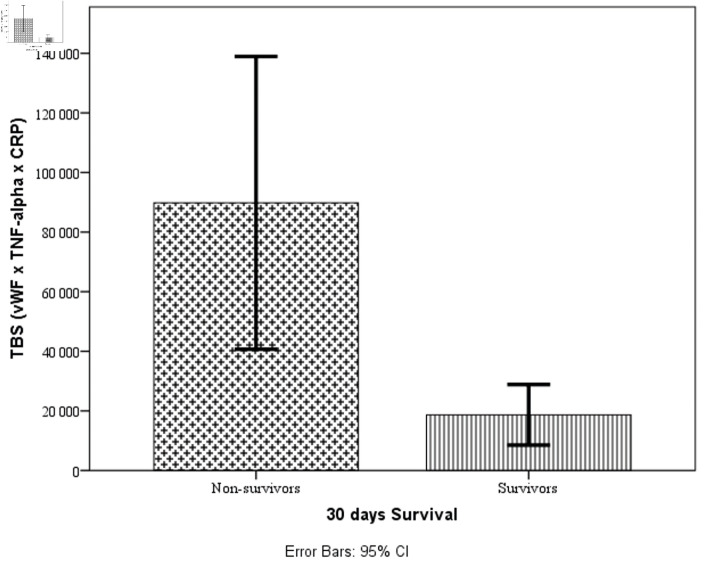
Box plots showing the distribution of TBS values between survivors and non-survivors. vWF: von Willebrand factor; TNF-α: tumor necrosis factor-α; TBS: triple biomarker score; CRP: C-reactive protein; CI: confidence interval.

The TBS (TBS = vWF × TNF-α × CRP) was calculated for each patient at admission. Figure 2 displays the distribution of TBS values between survivors and non-survivors using box plots.

**Figure 3:**
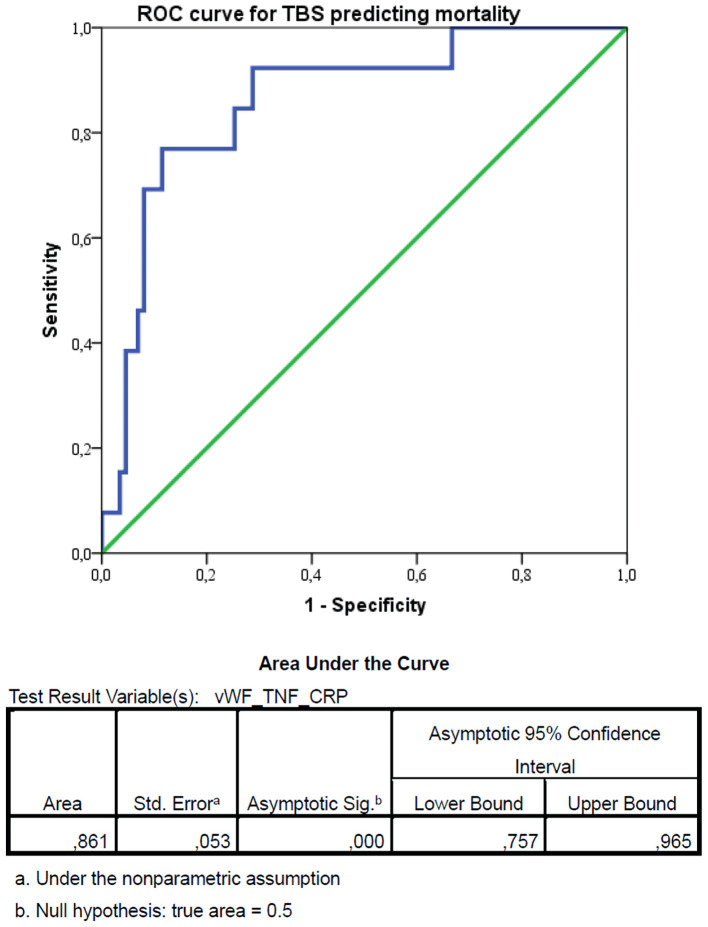
Receiver operating characteristic (ROC) curve for the triple biomarker score (TBS) predicting in-hospital mortality. The optimal cut-off value was identified as 4952.65, using Youden’s index maximization, which yielded a sensitivity of 92.3% and a specificity of 69.0% (Youden’s index J = 0.613). vWF: von Willebrand factor; TNF-α: tumor necrosis factor-α; CRP: C-reactive protein; CI: confidence interval.

ROC analysis revealed that the TBS had excellent discriminatory power for predicting in-hospital mortality, with an AUC of 0.861 (95% CI, 0.782–0.940; P < 0.001) (Fig. 3). Using Youden’s index maximization, the optimal cut-off value was identified as 4952.65, which yielded a sensitivity of 92.3% and a specificity of 69.0% (Youden’s index J = 0.613). Bootstrap validation confirmed the stability of these estimates (bootstrap 95% CI for AUC: 0.770–0.935). Patients with TBS above this cut-off had significantly lower survival rates in Kaplan–Meier analysis (69.2% vs. 98.4%, log-rank P < 0.001) and 26.7-fold higher odds of mortality in logistic regression analysis (odds ratio (OR) = 26.667; 95% CI, 3.298–215.586; p = 0.002).

Patients were stratified into high-risk and low-risk groups based on the optimal TBS cut-off value of 4952.65 identified by ROC analysis. Binary logistic regression was performed to evaluate the association between TBS and mortality, with mortality (1 = died, 0 = survived) as the dependent variable. As presented in Table 6, TBS was a significant predictor of mortality, with patients in the high TBS group having 26.7-fold higher odds of death compared to those in the low TBS group (OR = 26.667; 95% CI, 3.298–215.586; P = 0.002). The model demonstrated good fit (Hosmer-Lemeshow χ^2^ = 6.21, df = 8, P = 0.62) and explained 51% of the variance in mortality outcomes (Nagelkerke R^2^ = 0.51).

**Table 6:** Logistic Regression Analysis for Mortality Prediction

Variables in the equation								
		B	SE	Wald	df	Sig.	Exp(B)	95% CI for EXP(B) (lower–upper)
Step 1^a^	TBS	3.283	1.066	9.481	1	0.002	26.667	3.298–215.586
	Constant	–4.094	1.008	16.489	1	0.000	0.017	

^a^Variable(s) entered on step 1: TBS. Dependent variable: mortality (1 = died, 0 = survived). Model fit: Hosmer-Lemeshow χ^2^ = 6.21, df = 8, P = 0.62; Nagelkerke R^2^ = 0.51. TBS: triple biomarker score; SE: standard error; Sig.: significance; CI: confidence interval.

### Survival analysis

**Figure 4:**
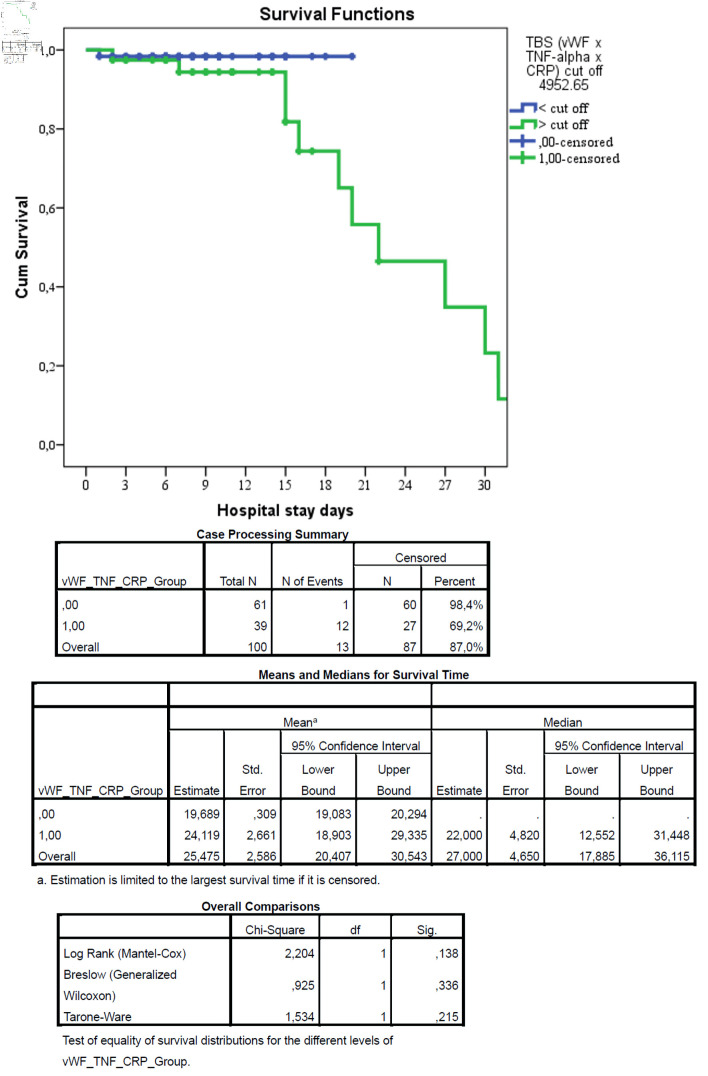
Kaplan–Meier survival curves for patients stratified by triple biomarker score (TBS). Kaplan–Meier survival analysis demonstrated no statistically significant difference between groups (log-rank test: χ^2^ = 2.204, P = 0.138). Although mortality was numerically lower in group 0 compared to group 1, the difference did not reach statistical significance, likely due to the limited number of events. Censored patients are indicated by tick marks. The low TBS group had significantly higher survival (98.4%) compared to the high TBS group (69.2%), with a log-rank P value < 0.001. The table below shows the number of patients at risk at each time point.

Kaplan–Meier survival analysis was performed to compare 30-day survival between the two risk groups. As shown in Figure 4, patients in the high-risk group (TBS > 4952.65) had significantly lower survival rates compared to those in the low-risk group (69.2% vs. 98.4%, log-rank P < 0.001). All mortality events occurred in patients with TBS above the cut-off value. This fact indicates that the high values of vWF × TNF-α × CRP interaction increased the risk of death.

## Discussion

### Principal findings

In this prospective cohort study of 100 patients with AP, we aimed to define the interactions between endothelial dysfunction and inflammation and to evaluate a novel triple-marker model combining vWF, TNF-α, and CRP. Our findings demonstrate a robust interplay between these pathways and establish the TBS (TBS = vWF × TNF-α × CRP) as a powerful predictor of mortality. The TBS achieved excellent discriminatory power with an area under the ROC curve of 0.861 (95% CI, 0.782–0.940; P < 0.001). Using Youden’s index maximization, we identified an optimal cut-off value of 4952.65, which yielded 92.3% sensitivity and 69.0% specificity for predicting mortality. Patients with high TBS (> 4952.65) had 26.7-fold higher odds of death (95% CI, 3.3–215.6; P = 0.002) and significantly lower 30-day survival (69.2% vs. 98.4%, log-rank P < 0.001) compared to those with low TBS. These results support the concept of a reciprocal inflammation–endothelial dysfunction axis in AP progression and suggest that combined biomarker panels provide superior prognostic accuracy compared to single-parameter models.

### The role of vWF in AP prognosis

Our finding that vWF is a critical component of the prognostic score aligns with a growing body of evidence implicating endothelial injury in AP pathogenesis. Li et al [14] demonstrated in 175 AP patients that vWF:Ag at admission was an independent risk factor for both severe AP (SAP) and death, and that dynamic monitoring of vWF:Ag changes further improved predictive value (AUC range 0.63–0.84). Similarly, Qin et al [15] studied 240 AP patients and found that vWF:Ag was significantly elevated in SAP compared to non-SAP cases, and that vWF:Ag independently predicted survival outcome (OR = 7.44; 95% CI, 1.24–44.82; P = 0.028). Their multivariate model combining vWF:Ag with the TyG index achieved an excellent AUC of 0.909 for prognosis prediction.

The mechanistic role of vWF in AP has been further elucidated by Chen et al [16], who measured endothelial markers in 57 AP patients and found that vWF levels correlated significantly with the extent of pancreatic necrosis and the development of multiple organ dysfunction syndrome (MODS). Notably, vWF showed the strongest association among the three endothelial markers studied (vWF, E-selectin, and endothelial protein C receptor (EPCR)). Morioka et al [17] provided additional pathophysiological insight by demonstrating that vWF:Ag was markedly elevated in SAP patients (402% of control values, P < 0.001) and remained persistently high in non-survivors, while gradually decreasing in survivors. They also identified an inverse correlation between vWF:Ag and ADAMTS13 activity, suggesting that impaired cleavage of ultra-large vWF multimers contributes to microvascular thrombosis and organ failure in SAP. This aligns with the established role of vWF in mediating platelet adhesion and aggregation under conditions of high shear stress, which may exacerbate pancreatic microcirculatory dysfunction [11].

### TNF-α as a driver of inflammation and organ dysfunction

TNF-α is recognized as a master pro-inflammatory cytokine in AP, and our results confirm its importance within the triple interaction. The strong correlations observed between TNF-α and endothelial markers (VEGF, endothelin, and E-selectin) on admission (r = 0.364–0.684, P < 0.001) confirm that cytokine-driven endothelial injury contributes significantly to disease severity. This is consistent with prior findings that proinflammatory cytokines induce endothelial adhesion molecules and TF expression, leading to platelet activation and microvascular thrombosis [8, 9].

Xiao et al [18] randomly assigned 120 SAP patients to conventional treatment with or without early blood purification and found that peak TNF-α levels were independent predictors of prognosis (OR = 1.02, P = 0.003), along with peak CRP and hemorheological parameters. Zhu et al [19] studied 32 patients with infected SAP and demonstrated that high TNF-α levels were associated with significant impairment of liver, kidney, and lung function, whereas patients with lower TNF-α levels mounted a more controlled inflammatory response. Arriaga-Pizano et al [20] measured cytokines in 15 AP patients and reported that TNF-α, IL-6, and IL-8 were significantly higher in non-survivors and in patients with APACHE II scores > 8, further supporting the prognostic value of pro-inflammatory cytokines. These observations are consistent with the well-established role of TNF-α in initiating the cytokine cascade that drives systemic inflammatory response syndrome (SIRS) and subsequent organ failure in SAP [9].

### CRP as the cornerstone of systemic inflammation assessment

CRP remains the most widely studied and clinically accessible marker of systemic inflammation in AP, and our study confirms its essential role within the triple biomarker panel. Shi et al [21] analyzed 463 AP patients and identified CRP measured within 48 h (CRP_48_) as an independent risk factor for SAP (P < 0.001), with an AUC of 0.802. When combined with calcium and the prognostic nutritional index (PNI), the AUC increased to 0.892, and the combination of PNI and Ranson score achieved an AUC of 0.936. Xu et al [22] conducted a large-scale study of 5,016 AP patients and introduced the C-reactive protein–albumin–lymphocyte (CALLY) index. They found that lower CALLY (indicating higher CRP relative to albumin and lymphocytes) was associated with increased SAP risk (adjusted OR = 0.49 per standard deviation (SD) increase, 95% CI, 0.32–0.76), and that CALLY (AUC = 0.764) outperformed traditional inflammatory markers including neutrophil-to-lymphocyte ratio (NLR), platelet-to-lymphocyte ratio (PLR), and systemic immune-inflammatory index (SII).

The close association between CRP and procalcitonin observed in our correlation analysis (r = 0.433 on admission, r = 0.555 on day 3) further supports the concept that systemic inflammation and secondary infection-related processes may coexist and amplify one another, forming a self-sustaining cycle of inflammation and tissue injury. This is consistent with the findings of Peng et al [23], who reviewed predictive factors in hypertriglyceridemic AP and identified CRP, calcium, prothrombin time (PT), and D-dimer as key prognostic markers.

### The emerging paradigm of combined biomarker panels

A consistent theme across recent literature is that combination models outperform individual markers. Huynh et al [24] prospectively studied 461 AP patients in Vietnam and developed the neutrophil × CRP index (NCI), which demonstrated excellent predictive performance for SAP (AUC = 0.853–0.897 across cohorts) and in-hospital mortality (AUC = 0.824–0.902). Notably, they identified a linear relationship between NCI and SAP risk, and the optimal cut-off for mortality prediction was NCI ≥ 3,180. Xie et al [25] evaluated 247 patients with hyperlipidemic AP and found that the CRP-to-lymphocyte ratio was an independent predictor of poor prognosis (hazard ratio (HR) = 2.05; 95% CI, 1.62–2.60). The combination of triglyceride glucose–body mass index, BISAP score, and CRP-to-lymphocyte ratio yielded an extraordinary AUC of 0.987 for predicting 28-day poor prognosis.

Our TBS aligns with this emerging paradigm. While Qin et al [15] combined vWF:Ag with the TyG index, and Huynh et al [24] combined neutrophils with CRP, our study is the first to our knowledge to integrate vWF (endothelial activation), TNF-α (proximal inflammation), and CRP (downstream inflammation) into a single multiplicative score. The biological rationale for this combination is strong: vWF reflects endothelial injury and microvascular thrombosis, TNF-α drives the initial cytokine cascade, and CRP captures the overall inflammatory burden. Their multiplicative interaction may capture synergistic effects that linear combinations cannot, as suggested by the fact that the three-way interaction remained significant in logistic regression while individual main effects did not.

The close association between VEGF and TNF-α observed in this study (r = 0.364, P < 0.001) suggests concurrent activation of inflammatory and angiogenic pathways. VEGF, while promoting endothelial repair, also increases vascular permeability through its interaction with VEGFR-2 receptors [10], thereby aggravating edema and ischemia in pancreatic tissue. Although vWF showed weaker individual correlations with inflammatory markers, its predictive value was significant when incorporated into the TBS. This suggests that the predictive power of vWF lies not in its absolute level alone, but in its synergistic relationship with TNF-α and CRP—a finding that echoes the work of Morioka et al [17], who emphasized the importance of the ADAMTS13/vWF axis in SAP pathogenesis.

### Comparison with existing prognostic scores

Traditional scoring systems for AP, including Ranson, BISAP, APACHE II, and CTSI, have well-documented limitations including complexity, delayed availability, and modest predictive accuracy [6, 5]. Recent studies have sought to identify simpler, laboratory-based alternatives. Shi et al [21] reported that BISAP (AUC = 0.895) and CTSI (AUC = 0.931) performed well in their cohort, but these scores require clinical and radiological data that may not be immediately available. In contrast, our TBS is derived entirely from admission blood tests and can be calculated automatically by hospital laboratory systems, providing clinicians with an immediate, quantitative risk score at the time of admission.

The performance of our TBS (AUC = 0.861) compares favorably with other recently proposed biomarkers. Huynh et al [24] reported AUCs of 0.853–0.897 for their NCI in predicting SAP, while Qin et al [15] achieved an AUC of 0.909 with their combined vWF/TyG model. Xu et al [22] reported an AUC of 0.764 for the CALLY index alone, which increased to 0.892 when combined with other markers. Our TBS thus falls within the range of these contemporary prognostic tools, with the added advantage of capturing the pathophysiological synergy between endothelial dysfunction and inflammation.

### Study strengths and limitations

The strengths of this study include its prospective design, the serial measurement of multiple biomarkers reflecting both inflammatory and endothelial pathways, and the integration of advanced statistical models including interaction analysis, ROC curve analysis with Youden’s index optimization, and Kaplan–Meier survival analysis with log-rank testing. The inclusion of both endothelial and inflammatory markers within a single analytical framework captures the multifactorial nature of AP progression, where vascular dysfunction, systemic inflammation, and coagulopathy converge to drive organ failure [12, 13].

Nevertheless, several limitations must be acknowledged. First, the single-center design and moderate sample size (n = 100, with 13 mortality events) may limit generalizability. While our findings are consistent with larger studies [14, 15, 21, 22], multicenter validation is required. Second, the wide CI for the OR (3.3–215.6) reflects the relatively small number of events and warrants cautious interpretation; this is comparable to the CIs reported by Qin et al [15] (OR = 7.44; 95% CI, 1.24–44.82). Third, we did not perform long-term follow-up beyond 30 days, so the utility of TBS for predicting late complications or long-term mortality remains unknown. Fourth, although we adjusted for key covariates including age, sex, and etiology, residual confounding cannot be excluded. Fifth, while our findings align with studies examining vWF [14–17], TNF-α [18–20], and CRP [21–25] individually, the multiplicative interaction model requires further validation in independent cohorts before clinical implementation.

The imbalance between mild (n = 68) and severe (n = 32) groups reflects the natural epidemiology of AP but may introduce bias. While we did not perform statistical adjustment for this imbalance due to the sample size, the primary mortality analysis focused on the TBS as a continuous variable rather than group comparisons. Future studies with larger cohorts should include propensity score matching or multivariate adjustment to confirm these findings.

### Clinical implications and future directions

Despite these limitations, our findings have potential clinical implications. Early identification of patients with elevated TBS could guide more intensive monitoring, earlier ICU admission, and timely therapeutic interventions. The TBS could be calculated automatically by hospital laboratory systems, providing clinicians with a simple, quantitative risk score at admission to aid in triage and management decisions.

Furthermore, therapies aimed at stabilizing endothelial function, modulating cytokine activity, or targeting the coagulation–inflammation interface may be particularly beneficial in patients with high TBS. Xiao et al [18] demonstrated that early blood purification (continuous veno-venous hemodiafiltration) significantly reduced CRP, TNF-α, and IL-6 levels while improving hemorheological parameters and clinical outcomes in SAP patients. Morioka et al [17] suggested that ADAMTS13 replacement could represent a therapeutic strategy for patients with severely reduced ADAMTS13 activity and accumulated ultra-large vWF multimers. Future studies should prospectively validate the TBS in multicenter cohorts, compare its performance against established scoring systems, and explore whether TBS-guided management improves clinical outcomes through randomized controlled trials.

### Conclusions

In conclusion, we demonstrate a robust interaction between endothelial dysfunction and systemic inflammation in AP. The novel TBS, integrating vWF, TNF-α, and CRP, provides superior prognostic accuracy for mortality (AUC = 0.861) and represents a practical tool for early risk stratification. Patients with TBS above the cut-off of 4952.65 have 26.7-fold higher odds of death and significantly lower 30-day survival. These findings underscore the critical role of the endothelial–inflammatory axis in determining AP outcomes and support the growing paradigm that combined biomarker panels outperform individual markers for prognosis prediction. External validation in larger, multicenter cohorts is warranted before widespread clinical implementation.

## Data Availability

The data supporting the findings of this study are available from the corresponding author upon reasonable request.
